# Geographical distribution of *MTHFR* C677T gene polymorphisms among the reproductive-age women in Chinese Han populations: based on migration

**DOI:** 10.1186/s12905-024-03244-3

**Published:** 2024-07-18

**Authors:** Yifen Shen, Yongchun Gu, Ying Tang, Hao Shen, Chao Liu

**Affiliations:** 1 Department of Central Lab, Suzhou Ninth People’s Hospital, Ludang Road 2666, Suzhou, Jiangsu Province 215200 China; 2 Department of Clinical Laboratory, Suzhou Ninth People’s Hospital, Ludang Road 2666, Suzhou, Jiangsu Province 215200 China

**Keywords:** Methylenetetrahydrofolate reductase, Polymorphism, Geographical distribution, Chinese Han populations, Migration

## Abstract

**Background:**

Methylenetetrahydrofolate reductase (MTHFR) is essential for the metabolism of folic acid and homocysteine. The *MTHFR* C677T polymorphism is associated with several disorders. Our study aims to explore the geographical distributions of the *MTHFR* C677T polymorphism of women in China and how migration affected the polymorphism in Suzhou.

**Methods:**

A total of 7188 women of reproductive age were recruited in Suzhou of the study. Subjects were classified according to their native places after data extraction. *MTHFR* C677T gene polymorphisms were detected by quantitative PCR with genomic DNA isolated from blood samples.

**Results:**

The frequencies of the 677T allele and 677TT genotype were higher in northern China than that in southern China and decreased in geographical gradients from north to south. The frequencies were considerably higher in the migrant population than that in the indigenous population of Suzhou. The migrant population have gradually changed the prevalence in Suzhou.

**Conclusions:**

Our study suggested that the prevalence of *MTHFR* C677T polymorphisms among women varied across different geographical regions in Chinese Han populations. The 677T allele frequencies of the northern populations were significantly higher than those of the southern populations. The migrant population gradually changed the prevalence of the *MTHFR* C677T polymorphism in Suzhou.

**Supplementary Information:**

The online version contains supplementary material available at 10.1186/s12905-024-03244-3.

## Background

Birth defects are a serious public health and societal problem in China. Approximately 900,000 neonates are born with birth defects annually, with an incidence of 5.6% [1]. The incidence of perinatal birth defects has increased in the last decade. Premarital health care and counseling is a critical component of maternal and child health, improving pregnancy outcomes and reducing birth defects and postpartum disease rates [2, 3].

Folic acid, also known as vitamin B9, must be obtained from diet and supplements, which is required for DNA replication and protein metabolism [4, 5]. It is now well-recognized that the consumption of folic acid prior to conception can effectively reduce birth defects, including neural tube defects (NTDs) [3, 4]. The health authorities issued guidelines [6] that recommended a daily folic acid addition of 0.4 to 0.8 mg for women of childbearing age. The recent concept of personalized medicine demands that dose selection must be based on the physiological/pathological conditions of each individual [7, 8].

Methylenetetrahydrofolate reductase, encoded by the *MTHFR* gene, is a critical regulatory enzyme engaged in the metabolism of homocysteine and the folate cycle, and it converts folic acid to tetrahydrofolic acid [9, 10]. It has been confirmed that the *MTHFR* gene variants C677T and A1298C are associated with low enzyme activity [11]. Decreased enzyme activity results in elevated intracellular homocysteine, which is implicated in increased risk for a variety of disorders, including central nervous system dysfunction, birth defects, and pregnancy complications [12, 13]. The C677T polymorphic site is characterized by a change of C into T at position 677 of the *MTHFR* gene (located 1p36.3). At codon 222, an alanine to valine substitution resulted in thermolability and reduced enzyme activity. Mutant homozygote (TT genotype) attenuates enzyme activity by 65% and heterozygote (CT genotype) by approximately 40% [14]. Therefore, detecting the genotype of each individual, as well as understanding the country-wide distribution is crucial for individual risk assessment, preventive medicine, and precision medicine.

Epidemiological studies have reported that the prevalence of the *MTHFR* C677T polymorphism varied apparently across different geographical distributions and that the frequency of *MTHFR* 677T allele is higher in Europe than that in Africa. It shows an increasing trend from north to south among Europeans and North Americans [11, 15–17]. In China, it shows a reverse trend [18], where a higher frequency was more likely detected in the northern provinces. Several studies performed among Chinese populations show distributional differences [11, 19]. Interestingly, the distribution of the proportion of several disorders, such as recurrent abortion, preeclampsia [20, 21], chromosomal aneuploidy [22] and NTDs [23], is similar to the *MTHFR* C677T gene distribution in China. Higher frequency of *MTHFR* 677T allele correlates with increased proportion of these disorders. Taking NTDs in China as an example, the prevalence is higher in the north than in the south (4.5‰ and 1.1‰, respectively) [24]. Additionally, Both the plasma folate concentrations and daily dietary folate intake of women in the north were lower than those of women in the south [25–27]. These data indicated that there are inner associations among *MTHFR* C677T gene distribution, folic acid intake and several disorders. These associations are complex and delicate, arising from long-term interactions between heredity and the environment.

Previous studies on geographical distributions of gene polymorphisms usually required collection of data from a large sample of subjects across various regions, which might be laborious and time-consuming, and demanded collaboration across multiple research teams. In our study, we took advantage of the demographic characteristics of Suzhou city, where our hospital located in and the study was carried out. Suzhou is a typical city of migrants. Benefitting from the booming local economy and increasing employment opportunities, approximately half of the population migrated from other cities or provinces in recent decades. It is convenient for our laboratory to obtain biological samples of subjects from various regions across China. We detected and evaluated the prevalence of *MTHFR* C677T polymorphisms among women of reproductive age residing in Suzhou (including migrants from other cities or provinces) and tried to elucidate its geological distribution pattern across China. Our study showed the geographical distribution pattern in a different way, which may help the government understand the trend of changes of the prevalence and formulate dynamic risk assessments.

## Methods

### Study population

The subjects were randomly selected reproductive-age women who had received inpatient or outpatient medical care in the Dept. of Obstetrics & Gynecology, Suzhou Ninth People’s Hospital, between July 2017 and December 2019. The inclusion criteria were as follows: (I) subjects were all native Chinese women; (II) subjects were aged 18–45 years; (III) subjects came for pre-pregnancy care or pregnancy care; (IV) subjects were healthy without basic diseases; (V) subjects could offer valid personal information not restricted to age, ethnic background, place of birth, or native place; and (V) subjects were required to have the *MTHFR* (C677T) gene polymorphism detected to guide folic acid addition or risk assessment.

After data extraction, a total of 7188 Han subjects were recruited from the Suzhou Ninth People’s Hospital in this study (mean age was 29.9 ± 6.7 years). Inquiries of census register ground was needed to attenuate the impact of migrants. All subjects were grouped into zones according to their own native places. Additionally, subjects were stratified into binary groups, the northern populations and the southern populations. According to previous studies, the divisional line was set at the Qinling Mountains-Huaihe River. To evaluate the impact of migration, subjects were divided into an indigenous population and a migrant population.

The study was performed in accordance with the Declaration of Helsinki, and the Ethics Committee of the Suzhou Ninth People’s Hospital granted ethical approval for the study (approval number: KY2022-100-01). Informed consent was acquired from all participants and subsequently anonymized.

### Genotyping

*MTHFR* C677T gene polymorphisms were detected by quantitative PCR. Genomic DNA was extracted from blood, using the TIANamp Blood DNA Kit according to the manufacturer’s instructions. (TIANGEN, CN). The assays were carried out with a TaqMan PCR Core Reagent Kit (ABI, USA). The primers and probes listed below (ABI, USA) were used [11]: for: *5’-GAAAAGCTGCGTGATGATG-3’*, rev: *5’-TTGAAGGAGAAGGTGTC-3’*, probe 1 (VIC-dye labeled): *AATCGGCTCCCGC*, probe 2 (FAM-dye labeled): *AATCGACTCCCGC*. PCR amplifications consisted of an initial step of 95 °C for 2 min and 45 cycles of 95 °C for 15 s and 60 °C for 1 min.

### Statistical analysis

All statistical analyses were performed using R 4.2.0 and the level of statistical significance considered was a *p* value below 0.05 unless otherwise stated. The 95% CIs of allelic and genotypic frequencies were calculated using the normal approximation. χ^2^ testing was performed to calculate Hardy-Weinberg equilibrium among the observed genotype frequencies. χ^2^ testing was performed to test the differences in allelic and genotypic frequencies between the northern and southern populations.

## Results

### Hardy-weinberg equilibrium

The genotypic distribution of the *MTHFR* C677T polymorphism was consistent with Hardy-Weinberg equilibrium, except for the *MTHFR* C677T polymorphism among the population from Henan Province.

### Impact of migration on frequencies

The subjects were divided into 2 major categories (migrant and indigenous) according to their registered native places. Balance diagnostics after propensity score matching (PSM) showed that the groups were comparable and other non-treatment factors were excluded (Supplementary Table 1). We found that the total pooled frequencies of the 677T allele and 677TT genotype were significantly higher in the migrant population (47.89% and 24.34%, respectively) than that in the indigenous population (37.62% and 14.36%, respectively) (*p* < 2.2 × 10^–16^) (Table 1).

**Table 1:** Distribution of *MTHFR* C677T polymorphism between indigenous population and migrant population

group	number of subjects	genotype (No.) and frequencies (%)									T allele frequency (%)	
		CC			CT			TT			T	
		No.	frequency	95% CI	No.	frequency	95% CI	No.	frequency	95% CI	frequency	95% CI
Indigenous^a b^	2709	1060	39.13	37.31–40.98	1260	46.51	44.64–48.39	389	14.36	13.09–15.73	37.62	36.34–38.92
migrant	4479	1279	28.56	27.26–29.90	2110	47.11	45.65–48.57	1090	24.34	23.11–25.62	47.89	46.86–48.93
**total**	7188	2339	32.54	31.47–33.63	3370	46.88	45.73–48.03	1479	20.58	19.64–21.51	44.02	43.21–44.83

*MTHFR*, methylenetetrahydrofolate reductase; CI, confidence interval; CC, ‘‘wild-type’’ homozygosity; CT, heterozygosity; TT, mutant homozygosity

^a^The 677TT genotype frequencies were significantly different from the migrant populations (χ2 = 102.80, *p* < 2.2 × 10^− 16^)

^b^The 677T allele frequency was significantly different from the migrant populations (χ2 = 144.64, *p* < 2.2 × 10^− 16^)

### Geographical distribution of the *MTHFR* C677T polymorphisms

**Fig. 1:**
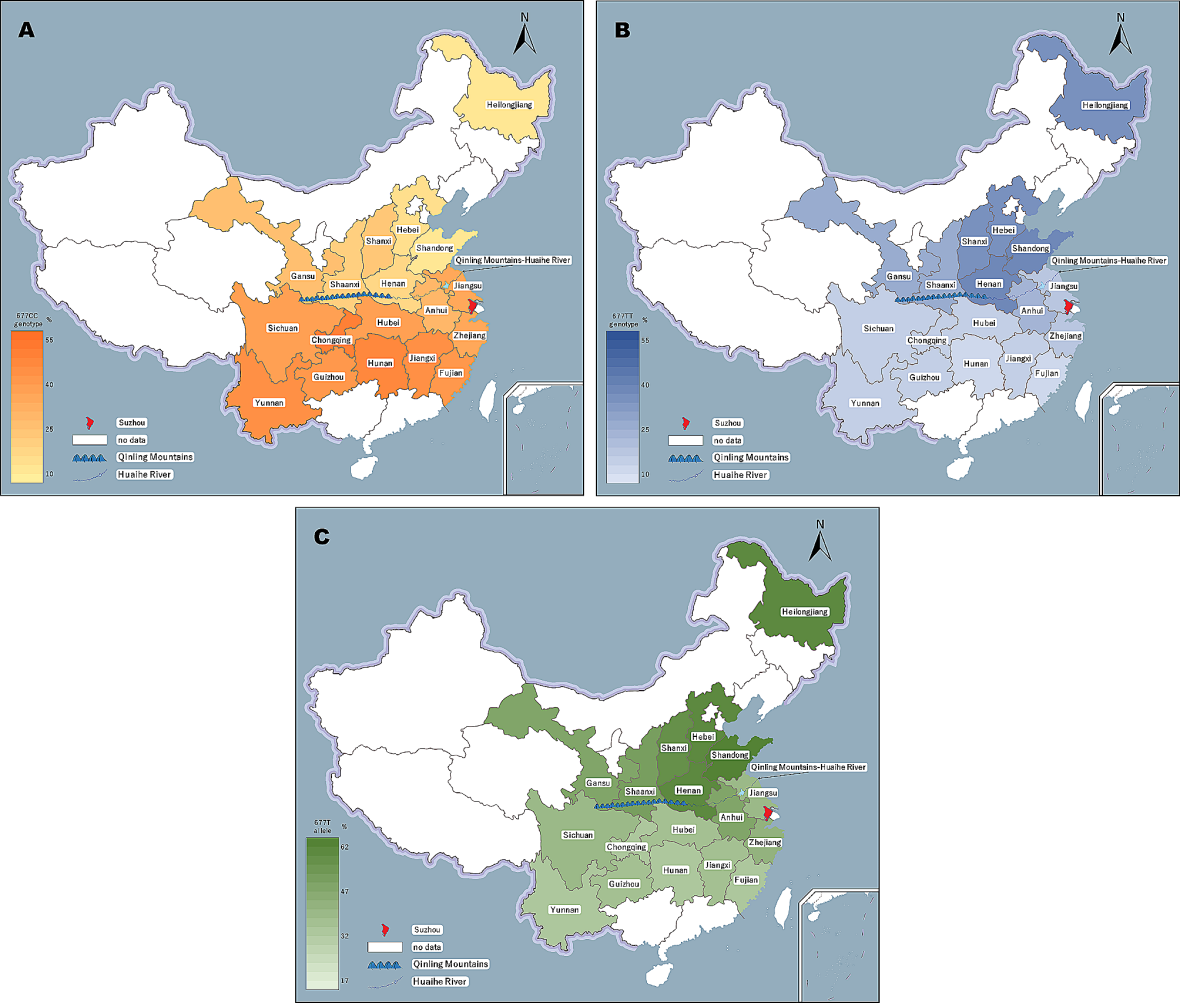
Map of China showing the geographical distributions of the *MTHFR* C677T polymorphisms in different regions. (**A**) The frequencies of the 677CC genotype increased in geographical gradients from north to south. The populations of Guangdong carried the highest frequencies of 677CC genotype. (**B**) The frequencies of the 677TT genotype decreased in geographical gradients from north to south. A similar pattern was recorded for the frequencies of 677T allele (**C**). Original map of China downloaded from website of Ministry of Natural Resources of the People’s Republic of China (http://bzdt.ch.mnr.gov.cn/browse.html?picId=“4o28b0625501ad13015501ad2bfc0696”)

**Fig. 2:**
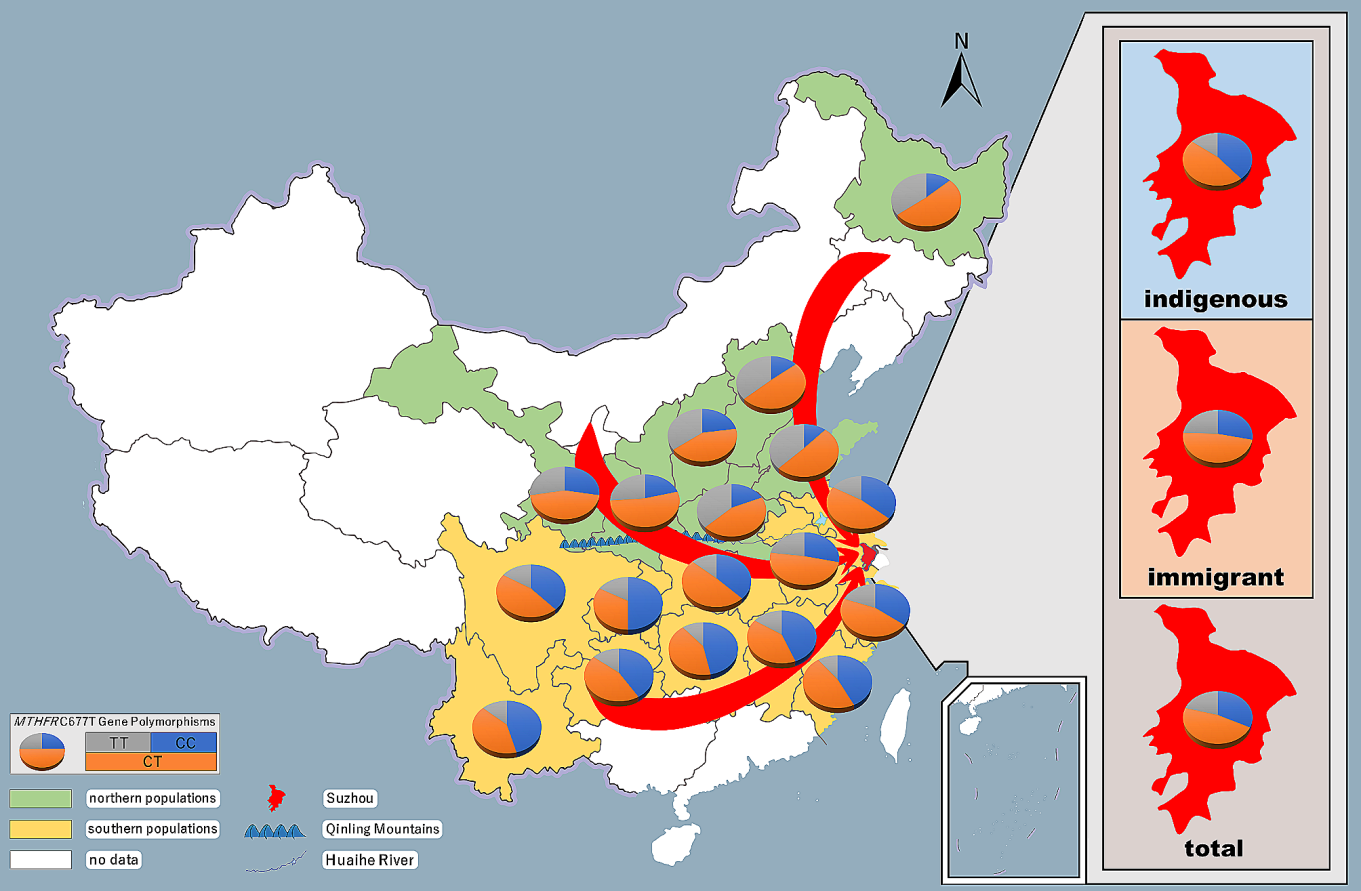
Impact of migrant population on the prevalence of the *MTHFR* C677T polymorphism in Suzhou. The northern population who carried higher frequency of the 677T allele, and the southern population who carried lower frequency of the 677T allele migrated to Suzhou, which changed the prevalence of the *MTHFR* C677T polymorphism in Suzhou (right panel)

The geographical distribution of the population prevalence of the *MTHFR* C677T polymorphisms is shown in Table 2; Fig. 1. The subjects were distributed across 18 provinces/municipalities. According to our data, the proportion of the 677T allele and the 677TT genotype varied among 18 distinct zones. It is suggested that the frequencies of the 677T allele and the 677TT genotype decreased in geographical gradients from north to south (Fig. 1), which were high in those northern provinces such as Heilongjiang, Hebei, Shanxi, Shandong, Henan and then decreased to an intermediate level of 20 − 30% in Gansu, Shannxi and Anhui (Anhui is the only southern population in this group) and were low in the remaining southern provinces/municipalities. Generally, the frequencies of the 677T allele and the 677TT genotype were significantly higher in North China than that in South China (*p* < 2.2 × 10^–16^). The heterogeneity of the migrant population changed the prevalence of the *MTHFR* C677T polymorphism in Suzhou in decades (Table 1; Fig. 2).

**Table 2:** Distribution of *MTHFR* C677T polymorphism among populations from 18 regions in China

group		number of subjects	genotype (No.) and frequencies (%)									T allele frequency (%)^b^	
			CC			CT			TT^a^			T	
			No.	frequency	95% CI	No.	frequency	95% CI	No.	frequency	95% CI	frequency	95% CI
**northern**	Heilongjiang	70	9	12.86	6.92–22.67	36	51.43	39.96–62.76	25	35.71	25.50-47.41	61.43	53.16–69.09
	Heibei	65	9	13.85	7.46–24.27	32	49.23	37.46–61.08	24	36.92	26.23–49.07	61.54	52.96–69.46
	Shannxi	288	60	20.83	16.54–25.89	152	52.78	47.02–58.47	76	26.39	21.63–31.77	52.78	48.70-56.83
	Gansu	172	48	27.91	21.75–35.04	76	44.19	36.98–51.66	48	27.91	21.75–35.04	50.00	44.75–55.25
	Shanxi	62	14	22.58	13.95–34.40	26	41.94	30.48–54.34	22	35.48	24.74–47.91	56.45	47.66–64.85
	Henan	634	116	18.30	15.41–21.58	281	44.32	40.50-48.21	237	37.38	33.70-41.21	59.54	56.81–62.21
	Shandong	219	25	11.42	14.66–29.18	111	50.68	44.10-57.23	83	37.90	31.74–44.48	63.24	58.63–67.62
**Southern** ^cd^	Jiangsu	3857	1410	36.56	35.05–38.09	1814	47.03	45.46–48.61	633	16.41	16.44–18.93	39.93	38.84–41.03
	Anhui	831	238	28.64	25.67–31.81	403	48.50	45.12–51.90	190	22.86	20.13–25.84	47.11	44.72–49.51
	Chongqing	42	21	50.00	35.53–64.47	14	33.33	21.01–48.44	7	16.67	8.32–30.61	33.33	24.17–43.94
	Hubei	227	86	37.89	31.83–44.35	111	48.90	42.47–55.37	30	13.22	9.42–18.24	37.67	33.33–42.21
	Hunan	128	60	46.88	38.45–55.49	54	42.19	33.98–50.85	14	10.94	6.63–17.53	32.03	26.62–37.98
	Sichuan	182	70	38.46	31.70–45.70	83	45.60	38.53–52.85	29	15.93	11.33–21.94	38.74	33.88–43.84
	Zhejiang	73	26	35.62	25.61–47.07	33	45.21	34.32–56.58	14	19.18	11.78–29.66	41.78	34.09–49.89
	Yunnan	61	28	45.90	34.01–58.27	25	40.98	29.53–53.50	8	13.11	6.79–23.80	33.61	25.84–42.38
	Jiangxi	136	60	44.12	36.05–52.51	55	40.44	32.56–48.84	21	15.44	10.33–22.45	35.66	30.20-41.52
	Guizhou	92	38	41.30	31.79–51.51	41	44.57	34.83–54.74	13	14.13	8.45–22.69	36.41	29.80-43.57
	Fujian	49	21	42.86	30.03–56.73	23	46.94	33.70-60.62	5	10.20	4.44–21.75	33.67	25.09–43.48
total		7188	2339	32.54	31.47–33.63	3370	46.88	45.73–48.05	1477	20.58	19.65–21.52	44.02	43.20-44.82

^a^The *677TT* genotype frequencies were significantly different among the 18 populations (χ^2^ = 268.72, *p* < 2.2 × 10^− 16^)

^b^The 677T allele frequency was significantly different among the 18 populations (χ^2^ = 379.37, *p* < 2.2 × 10^− 16^)

^c^The 677TT genotype frequencies were significantly different from the northern populations (χ^2^ = 214.13, *p* < 2.2 × 10^− 16^)

^d^The 677T allele frequency was significantly different from the northern populations (χ^2^ = 144.64, *p* < 2.2 × 10^− 16^)

## Discussion

Folic acid deficiency or excess can lead to various diseases. It is necessary to supplement folic acid in a rational way so that the supplemental dosage and the level after supplementation can be controlled within a reasonable range. Measures such as folic acid supplementation or food fortification should be further introduced to improve folic acid deficiency/insufficiency in certain high-risk folic acid deficient populations (northern populations in China, pregnant women, malnutrition, chronic alcohol use, chronic hemolytic anemia, genetic variation in genes of folate metabolism) [24].

Among the populations characterized by gene variants are mainly the *MTHFR* C677T genotype. It is encouraged that women (without high-risk factors) considering or planning a pregnancy take an oral daily dosage of 0.4–0.8 mg folic acid from pre-conception to 3-month gestation. Women with the *MTHFR* 677TT genotype can increase the supplementation dose or extend the pre-pregnancy supplementation time. According to the guideline for the prevention of NTDs, a supplement dosage of 0.8-1.0 mg folic acid throughout the pregnancy is recommended for women with high-risk factors. Notably, hyperhomocysteinemic patients with the *MTHFR* 677TT genotype should take higher doses of folic acid [28].

Patterns of the distribution of *MTHFR* C677T polymorphism may help us understand the prevalence status and formulate public health policies. In this study, we examined the prevalence of the *MTHFR* C677T polymorphism among a total of 7188 reproductive-age women residing in Suzhou and a large proportion of them migrated from other provinces. The geographical distribution of the *MTHFR* C677T polymorphism presented a wide and gradual pattern from south to north China.

Migration is a critical contributor to the Hardy-Weinberg equilibrium and may lead to frequency deviation of gene polymorphism. According to the sixth census data and the Suzhou Statistical Bulletin on National Economic and Social Development, the migrant population (6,538,536) of Suzhou has exceeded the indigenous population (6,538,372) since 2013 [29, 30]. Suzhou has been the second largest migrant city in China [31]. The number of newborns of the migrant population showed a rocketing trend from 2005 to 2009 and even exceeded that of the household population (46.2 thousand vs. 41.4 thousand) in 2008 [30]. It was suggested that approximately half of the pregnant women in Suzhou were migrants. After data extraction, the migrant population was almost 1.65-fold of the indigenous population (4479 vs. 2709) in the study. The differences in the population between our study and census data exist partly because of the definition of the indigenous population. It is worth noting that the “indigenous population” here refers to the population of individuals who were born in Suzhou. The “migrant population” consists of individuals who migrate to Suzhou, whether they own household registration in Suzhou or not. Actually, a considerable number of individuals have received household registration in Suzhou. Our data showed the frequencies of the 677T allele and the 677TT genotype in the migrant population, suggesting that the recent migration wave has imperceptibly changed the prevalence of the *MTHFR* C677T polymorphism in Suzhou. The frequencies of the 677T allele and the 677TT genotype were dramatically increased due to the influx of the migrant population from those high frequency regions (northern provinces).

To explore the country-wide distribution pattern of the *MTHFR* C677T polymorphism, native places were identified as the subject’s geological region in view of the fact that the genotype would not change after birth, and this can offset the impact of migration in the last 20 years. The spatial-temporal demographic evolution could significantly change the genetic polymorphism of a migrant city. Considering that the age of subjects was in the range of 18–45 years old, our data reflect a natural status of approximately 20 years ago.

Our data indicated that the frequencies of the 677T allele and the 677TT genotype varied widely in different regions. The 677T allele frequencies of the northern populations varied between 50.00% and 63.24%, which were significantly higher than those of the southern populations (32.03-47.11%). Furthermore, according to the statistics above, southern populations are more diversified than northern populations. These findings may be due to the diverse geographical characteristics and population histories. The geography and climate separatrix between the south and north separated two vastly different regions. As shown, the frequencies of the 677T allele and the 677TT genotype display a decreasing trend along latitude from north to south. Yang et al. reported apparent geographical trends in which the frequencies changed as an inverse V pattern in latitude and longitude [11]. A recent study reported a similar pattern that the frequencies of the 677T allele and the 677TT genotype had a significant north-south difference. the frequencies of T allele of Shandong, Henan and Hebei ranked at the top in China [20]. The authors suggested that the frequency of T allele was consistent with the geographical distribution of pregnancy complications.

In our study, the top three frequencies are in Hebei, Heilongjiang and Henan. The frequencies of the 677T allele and the 677TT genotype peaked in Shangdong (63.24% and 37.90%, respectively) and presented a decreasing pattern from east to west in northern China. Differing from published studies, we focus more on the impact of population migration on the distribution. Our data shows a dynamic trend that the frequencies of the 677TT genotype raised gradually due to the migration. This trend has its social significance that can provide concrete information to the Government for reference. For example, now government departments in Suzhou may increase publicity and education, increase the percentage of cases tested for the *MTHFR* genotype and vice versa. It suggests that estimating the frequency of a region through preliminary calculations of population inflows and outflows is a feasible way.

Previous scholars have reported that the frequencies of the 677T allele and the 677TT genotype also vary among ethnic groups in China. The mean frequency of the 677T allele of the Han population was lower than that of Tujia and higher than that of Duar, Man, She and several other ethnic groups [32]. In this study, 52 ethnic minority subjects from 12 other ethnic groups were recruited. Due to the widely scattered subjects and the small sample size, these subjects were not included in the study.

In addition to the *MTHFR* C677T gene polymorphism, there are numerous other variations giving rise to *MTHFR* alleles. A previous study reported 14 nonsynonymous changes, including 11 rare SNPs each with a frequency under 1% and 3 common SNPs (C677T, A1298C and G1793A), among which SNP C677T is thought to be the best-studied case [33]. *MTHFR* A1298C is a change of A to C at position 1298, leading to a decrease in enzyme activity, which has weaker effects than the C677T polymorphism. The frequencies of the *MTHFR* A1298C genotype varied considerably among different populations and showed an increasing trend from north to south [11, 13]. *MTHFR* G1793A is a G/A base transition at position 1793. The allele was associated with deficient values in folic acid [34]. Investigations of the distribution of *MTHFR* G1793A were not adequate. Overall, the frequencies of the *MTHFR* 1793 A allele were lower in northern than in southern China [35].

Uncertainty surrounds the causes of the geographic gradient. Publications have indicated that the polymorphism is influenced by genetic, dietary, environmental, and demographic variables. Reinventing human relocation and migration may also be responsible for the distributional pattern [11, 36–38]. It has come to a consensus that the historical, geographical and population diversity conditions of China bring challenges for individualized plans of folic acid supplementation. In conclusion, our study indicates that the prevalence of the *MTHFR* C677T polymorphisms varies considerably by region between Chinese Han populations. Our study intends to present a clear picture of the *MTHFR* C677T polymorphism and serve as a reference for folate intake and risk assessment for several associated anomalies. Our data can help the government formulate precise health care strategies.

## Conclusions

1. The total frequencies of the 677T allele and 677TT genotype were significantly higher in the migrant population than that in the indigenous population.
2. The 677T allele frequencies of the northern populations were significantly higher than those of the southern populations.
3. The prevalence of the *MTHFR* C677T polymorphism in Suzhou is being changed and influenced gradually by the demographic diversity of migrant populations.

### Electronic supplementary material

Below is the link to the electronic supplementary material.

Supplementary Material 1

## Data Availability

The datasets used and/or analyzed during the current study are available from the corresponding author on reasonable request.

## Abbreviations

MTHFR: Methylenetetrahydrofolate reductase
NTD: Neural tube defect
PSM: Propensity score matching

## References

[1] Liu Y, Yang LL, Xu SY, Zhao ZY (2018). Pediatrics in China: challenges and prospects. World J Pediatr.

[2] Barbosa PR, Stabler SP, Machado AL, Braga RC, Hirata RD, Hirata MH, Sampaio-Neto LF, Allen RH, Guerra-Shinohara EM (2008). Association between decreased vitamin levels and MTHFR, MTR and MTRR gene polymorphisms as determinants for elevated total homocysteine concentrations in pregnant women. Eur J Clin Nutr.

[3] Murthy GV, Kolli SR, Neogi SB, Singh S, Allagh KP, John N, Ramani NS, Shamanna S, Doyle BR (2016). A mixed-method study to determine the benefits of Periconceptional Folic Acid Supplementation and effects of Folic Acid Deficiency in Mothers on Birth outcomes. JMIR Res Protoc.

[4] Fenech M (2012). Folate (vitamin B9) and vitamin B12 and their function in the maintenance of nuclear and mitochondrial genome integrity. Mutat Res.

[5] Bailey LB, Gregory JF (1999). 3rd: Folate metabolism and requirements. J Nutr.

[6] Group TGP, Ren A, Zhang X, Liu H, Zhu L, Liu K, Jia Y (2017). Guideline for the Prevention of neural tube defects by Periconceptional Folic Acid supplementation (2017). Chin J Reproductive Health.

[7] Viswanathan M, Treiman KA, Kish-Doto J, Middleton JC, Coker-Schwimmer EJ, Nicholson WK (2017). Folic acid supplementation for the Prevention of neural tube defects: an updated evidence report and systematic review for the US Preventive Services Task Force. JAMA.

[8] Fischer M, Stronati M, Lanari M (2017). Mediterranean diet, folic acid, and neural tube defects. Ital J Pediatr.

[9] Fernandes SP, Kvitko K, da Silva J, Rohr P, Bandinelli E, Kahl VF, Mai C, Brenner N, da Silva FR (2017). Influence of vitamin intake and MTHFR polymorphism on the levels of DNA damage in tobacco farmers. Int J Occup Environ Health.

[10] Bagley PJ, Selhub J (1998). A common mutation in the methylenetetrahydrofolate reductase gene is associated with an accumulation of formylated tetrahydrofolates in red blood cells. Proc Natl Acad Sci USA.

[11] Yang B, Liu Y, Li Y, Fan S, Zhi X, Lu X, Wang D, Zheng Q, Wang Y, Wang Y (2013). Geographical distribution of MTHFR C677T, A1298C and MTRR A66G gene polymorphisms in China: findings from 15357 adults of Han nationality. PLoS ONE.

[12] Troen AM (2005). The central nervous system in animal models of hyperhomocysteinemia. Prog Neuropsychopharmacol Biol Psychiatry.

[13] Lajin B, Alhaj Sakur A, Michati R, Alachkar A (2012). Association between MTHFR C677T and A1298C, and MTRR A66G polymorphisms and susceptibility to schizophrenia in a Syrian study cohort. Asian J Psychiatr.

[14] Rozen R (1997). Genetic predisposition to hyperhomocysteinemia: deficiency of methylenetetrahydrofolate reductase (MTHFR). Thromb Haemost.

[15] Binia A, Contreras AV, Canizales-Quinteros S, Alonzo VA, Tejero ME, Silva-Zolezzi I (2014). Geographical and ethnic distribution of single nucleotide polymorphisms within genes of the folate/homocysteine pathway metabolism. Genes Nutr.

[16] Wilcken B, Bamforth F, Li Z, Zhu H, Ritvanen A, Renlund M, Stoll C, Alembik Y, Dott B, Czeizel AE (2003). Geographical and ethnic variation of the 677C > T allele of 5,10 methylenetetrahydrofolate reductase (MTHFR): findings from over 7000 newborns from 16 areas world wide. J Med Genet.

[17] Yafei W, Lijun P, Jinfeng W, Xiaoying Z (2012). Is the prevalence of MTHFR C677T polymorphism associated with ultraviolet radiation in Eurasia?. J Hum Genet.

[18] Yang B, Fan S, Zhi X, Xia R, Wang Y, Zheng Q, Sun G (2017). Geographical and ethnic distribution of MTHFR gene polymorphisms and their associations with diseases among Chinese population. Clin Genet.

[19] Cui H, Lu Y, Ma S, Xue Y, Wang T, Duan G, Yang Q (2015). [Geographical distribution of MTHFR and MTRR gene polymorphisms among the Han women in Zhengzhou city]. Zhong Nan Da Xue Xue Bao Yi Xue Ban.

[20] Lin H, Liao C, Zhang R (2023). Regional distribution of the MTHFR C677T polymorphism in Chinese females. Front Genet.

[21] Zeng H, He D, Zhao Y, Liu NG, Xie H (2021). Association between MTHFR polymorphisms (MTHFR C677T, MTHFR A1298C) and recurrent implantation failure: a systematic review and meta-analysis. Arch Gynecol Obstet.

[22] Oliveira KC, Bianco B, Verreschi IT, Guedes AD, Galera BB, Galera MF, Barbosa CP, Lipay MV (2008). Prevalence of the polymorphism MTHFR A1298C and not MTHFR C677T is related to chromosomal aneuploidy in Brazilian Turner Syndrome patients. Arquivos brasileiros de endocrinologia e Metabologia.

[23] Li X, Zhu J, Wang Y, Mu D, Dai L, Zhou G, Li Q, Wang H, Li M, Liang J (2013). Geographic and urban-rural disparities in the total prevalence of neural tube defects and their subtypes during 2006–2008 in China: a study using the hospital-based birth defects surveillance system. BMC Public Health.

[24] Nutrition and Metabolism of China Maternal and Child Health Association TCRUoDCoCMEATHBoCAfIEaPoHCTECoP (2020). Zhong Guo Lin Chuang He Li Bu Chong Ye Suan Duo Xue Ke Zhuan Jia Gong Shi. Chin J Front Med Science(Electronic Version).

[25] Meng Q, Zhang L, Liu J, Li Z, Jin L, Zhang Y, Wang L, Ren A (2015). Dietary folate intake levels in rural women immediately before pregnancy in Northern China. Birth Defects Res Part Clin Mol Teratology.

[26] Zhao Y, Hao L, Zhang L, Tian Y, Cao Y, Xia H, Deng Y, Wang T, Yu M, Li Z (2009). Plasma folate status and dietary folate intake among Chinese women of childbearing age. Matern Child Nutr.

[27] Ma R, Wang L, Jin L, Li Z, Ren A (2017). Plasma folate levels and associated factors in women planning to become pregnant in a population with high prevalence of neural tube defects. Birth Defects Res.

[28] Quere I, Mercier E, Bellet H, Janbon C, Mares P, Gris JC (2001). Vitamin supplementation and pregnancy outcome in women with recurrent early pregnancy loss and hyperhomocysteinemia. Fertil Steril.

[29] Can-ming C, Jin-jun D (2015). Study on the socio-spatial differentiation in Suzhou: Data analysis based on the sixth National Population Census. J Nanjing Univ Posts Telecommunications(Social Science).

[30] Yuan-tao L, Jun-lin L (2014). Suzhou Population Bearing Capacity and Population Control Study. J Changshu Inst Technology(Philosophy & Social Sciences).

[31] Group SJMWTRP. On the harmonious Flow of migrant workers in Suzhou:an analysis based on the Sixth National Census Data. J Suzhou Univ Sci Technol (Soc Sci). 2013;30(5).

[32] Mao R, Fan Y, Chen F, Sun D, Bai J, Fu S (2008). Methylenetetrahydrofolate reductase gene polymorphisms in 13 Chinese ethnic populations. Cell Biochem Funct.

[33] Marini NJ, Gin J, Ziegle J, Keho KH, Ginzinger D, Gilbert DA, Rine J (2008). The prevalence of folate-remedial MTHFR enzyme variants in humans. Proc Natl Acad Sci USA.

[34] Melo SS, Persuhn DC, Meirelles MS, Jordao AA, Vannucchi H (2006). G1793A polymorphisms in the methylene-tetrahydrofolate gene: effect of folic acid on homocysteine levels. Mol Nutr Food Res.

[35] Mao R, Fan Y, Chen F, Fu S (2008). Genetic polymorphism of MTHFR G1793A in Chinese populations. Eur J Epidemiol.

[36] Cordain L, Hickey MS. Ultraviolet radiation represents an evolutionary selective pressure for the south-to-north gradient of the MTHFR 677TT genotype. Am J Clin Nutr. 2006;84(5):1244–45. 10.1093/ajcn/84.5.1243 17093181

[37] Wang D, He Y, Li Y, Luan D, Yang X, Zhai F, Ma G (2011). Dietary patterns and hypertension among Chinese adults: a nationally representative cross-sectional study. BMC Public Health.

[38] Hao L, Ma J, Stampfer MJ, Ren A, Tian Y, Tang Y, Willett WC, Li Z (2003). Geographical, seasonal and gender differences in folate status among Chinese adults. J Nutr.

