# Supplementary material for: Geographical distribution of MTHFR C677T gene polymorphisms among the reproductive-age women in Chinese Han populations: based on migration
Source: BMC Womens Health. 2024 Jul 18;24:407. doi: 10.1186/s12905-024-03244-3 (PMC11256367; doi:10.1186/s12905-024-03244-3)
Supplement: Supplementary file 1 — Supplementary Material 1 [file 12905_2024_3244_MOESM1_ESM.docx]

**Supplementary Table 1. Comparison of baseline characteristics between migrant and indigenous categories in the PSM sample.**

| variable | sample | **migrant（average）** | **indigenous（average）** | **standardized differences（%）** | **reduction in differences before and after matching（%）** | **t** | ***p* value** |
| --- | --- | --- | --- | --- | --- | --- | --- |
| age | unmatched | 2.382 | 2.31 | 14.5 | 94.3 | 7.166 | <0.001 |
|  | matched | 2.382 | 2.378 | 0.8 |  | 0.392 | 0.695 |
| family^a^ | unmatched | 65.642 | 68.895 | 4.4 | 75.3 | -2.214 | 0.027 |
|  | matched | 65.642 | 66.502 | 1.1 |  | -0.526 | 0.599 |
|  | | | | | | | |

PSM showed that the balance of baseline covariates between migrant and indigenous populations.

^a^ Sorted by individuals’ family name.
